# Effect of Micromixer Design on Lipid Nanocarriers Manufacturing for the Delivery of Proteins and Nucleic Acids

**DOI:** 10.3390/pharmaceutics16040507

**Published:** 2024-04-07

**Authors:** Enrica Chiesa, Alessandro Caimi, Marco Bellotti, Alessia Giglio, Bice Conti, Rossella Dorati, Ferdinando Auricchio, Ida Genta

**Affiliations:** 1 Department of Drug Sciences, University of Pavia, V.le Taramelli 12, 27100 Pavia, Italy; enrica.chiesa@unipv.it (E.C.); alessia.giglio@iusspavia.it (A.G.); bice.conti@unipv.it (B.C.); rossella.dorati@unipv.it (R.D.); 2 Department of Civil Engineering and Architecture, University of Pavia, Via Ferrata 3, 27100 Pavia, Italy; alessandro.caimi@unipv.it (A.C.); marco.bellotti01@universitadipavia.it (M.B.)

**Keywords:** liposomes, lipid nanoparticles, lipid nanocarriers, manufacture, microfluidics, nucleic acids, proteins

## Abstract

Lipid-based nanocarriers have emerged as helpful tools to deliver sensible biomolecules such as proteins and oligonucleotides. To have a fast and robust microfluidic-based nanoparticle synthesis method, the setup of versatile equipment should allow for the rapid transfer to scale cost-effectively while ensuring tunable, precise and reproducible nanoparticle attributes. The present work aims to assess the effect of different micromixer geometries on the manufacturing of lipid nanocarriers taking into account the influence on the mixing efficiency by changing the fluid–fluid interface and indeed the mass transfer. Since the geometry of the adopted micromixer varies from those already published, a Design of Experiment (DoE) was necessary to identify the operating (total flow, flow rate ratio) and formulation (lipid concentration, lipid molar ratios) parameters affecting the nanocarrier quality. The suitable application of the platform was investigated by producing neutral, stealth and cationic liposomes, using DaunoXome^®^, Myocet^®^, Onivyde^®^ and Onpattro^®^ as the benchmark. The effect of condensing lipid (DOTAP, 3–10–20 mol%), coating lipids (DSPE-PEG550 and DSPE-PEG2000), as well as structural lipids (DSPC, eggPC) was pointed out. A very satisfactory encapsulation efficiency, always higher than 70%, was successfully obtained for model biomolecules (myoglobin, short and long nucleic acids).

## 1. Introduction

Therapeutic approaches have expanded beyond small molecules to include nucleic acids, peptides, proteins and antibodies, and, over the last few years, biomolecules have gained considerable attention with a large number in clinical pipelines as vaccines or treatment of several different diseases [1]. However, these molecules are highly unstable in the physiological environment, since they undergo a rapid degradation process driven by proteases and ribonucleases. Moreover, their physical characteristics such as anionic charge and high molecular weight hamper the passive diffusion across the cell membranes as well as endosomal escape in the cytosol [2]. To mitigate these setbacks, drug delivery technologies were adapted to address the new challenges and nanocarriers have been studied to protect the payload from premature degradation and to enhance drug retention in the target site [2,3,4,5]. Among these, lipid-based nanocarriers are often the preferred choice for carrying sensible biomolecules since they naturally interact with cell membranes, and they have shown less risk of undesirable immunogenic effects [2,3,6]. Liposomes, self-assembled vesicles based on lipid bilayers enclosing an aqueous core, are the earliest version of lipid-based nanocarriers and they are widely exploited for biomedical and biotechnological purposes. Several clinically significant liposomal drug products (i.e., DaunoXome^®^, Myocet^®^, Onivyde^®^ and Vyxeos^®^) have been approved and released for different pathologies in the last 25 years [7]. Next generations of lipid nanocarriers, namely lipid nanoparticles (LNPs), nanostructured lipid carriers and cationic lipid–nucleic acid complexes, show more complex structures and enhanced physical stability [6]. In particular, RNA-loaded LNPs comprise stable complexes between synthetic cationic lipids and anionic nucleic acids, which are condensed in the core of the LNPs. Thanks to their ability to encapsulate and deliver therapeutics into the desired site within the body at a desired time, LNPs have successfully entered the clinic for the delivery of RNAs [6,7]. Within this paper, we refer to LNPs when complexation between cationic lipid and nucleic acid is present; in any other case, we refer to liposomes.

Since the clinical value of lipid nanocarriers is growing along with personalized medicine therapeutic approaches, the need for strongly reproducible production technology for liposomes as well as LNPs is challenging, making nanocarriers manufacturing a paramount research topic worldwide [8,9]. Most common traditional bulk methods to produce liposomes and LNPs, such as lipid film hydration and ethanol injection, yield large and heterogeneous nanoparticle populations on account of the poor control of the reaction environment and, hence, the lipids assembly [8]. Therefore, these methods require further size reduction methods, i.e., extrusion, sonication [10]. Present and future manufacturing challenges become opportunities for innovations and new technologies. In this framework, microfluidic devices, designed to tune reaction conditions by controlling fluids diffusion rate and pattern, are revolutionizing the lipid nanocarrier formulation and production by ensuring highly reproducible continuous manufacturing [11,12]. Recently, several microfluidic devices have been developed and tested for the manufacture of LNPs with outstanding results, given that several FDA/EMA-approved liposomal formulations (i.e., Onpattro^®^) employ such devices [13]. We can further hypothesize that soon microfluidics will become the gold standard for the production of liposomes, as well as LNPs, and new platforms need to be developed and customized to face the increasingly demanding product attributes (i.e., surface decoration, multitargeting features). Moreover, the expansion of continuous flow synthesis through microfluidics is not limited to the production of polymeric and lipidic nanocarriers but also it can be successfully utilized for bioconjugation reactions to obtain drug–antibody conjugates paving the way to new targeted therapeutics [14].

In this work, a brand new microfluidic platform for the NPs production was fabricated with the aim of improving the configuration flexibility to a wide range of cartridges and consumables easily available on the market and covering different pharmaceutical applications as well as reducing the formulation screening process and manufacturing process costs.

Even if the micromixer seems similar to other devices available on the market, slight differences in the microchannel geometry induce large differences in the mixing. In static micromixers, the mixing of solvent and anti-solvent is driven by a non-turbulent chaotic fluid dynamic resulting from sudden alterations in a cross-sectional area, like those seen in staggered herringbone structures [15]. On the contrary, in diffusive micromixers, the formation of nanoparticles is facilitated by the molecular diffusion of the active ingredient (e.g., polymer chain or lipid unit) between the two fluids. As a result, the cartridge needs to compress the two fluids. Nevertheless, in each static micromixer, the secondary flows can be influenced by different structural geometrical features such as the size of the channel cross-section, the changing of the cross-section along the chip length and the presence of bending and curvature. Different designs lead to a different fluid dynamic and a different mixing while keeping the same working conditions. Hence, the adoption of different microfluidic cartridges involves a new optimization of the manufacturing process [16].

Hence, the aim of the present work is to test the reliability of the microfluidic manufacturing of lipid nanocarriers (both liposomes and LNPs) through an in-house developed microfluidic device. Although several works reported the optimization of the process to produce liposomes or LNPs, the effects on the lipid nanocarriers of chaotic micromixers different from the platforms available on the market contribute to the novelty of the present work.

Taking advantage of configuration flexibility without sacrificing the high throughput and accuracy of the mixing, the effect of different microfluidic cartridges on the nanocarriers production was assessed by producing neutral, stealth and cationic liposomes by using as a reference the materials and composition of commercial liposomal products.

To investigate various operating and formulation parameters influencing the critical quality attributes of the final formulation, a systematic Quality by Design approach was applied. Moreover, model biomolecules with different chemistry, geometry and molecular weights were selected to evaluate the delivery ability of nanocarriers produced by the microfluidic device. Myoglobin is a globular protein with a molecular weight of 17 kDa, whereas negative control siRNA and single-stranded DNA (ssDNA) were used as model nucleic acids to obtain LNPs after complexation with the cationic lipid. Negative control siRNA is a double-stranded RNA of 21 nucleotides; instead, ssDNA is a single long chain of 587–831 bases.

## 2. Materials and Methods

### 2.1. Materials

1,2-distearoyl-sn-glycero-3-phosphocholine (18:0 PC—DSPC, Mw 790.15 g/mol), 1,2-distearoyl-sn-glycero-3-phosphoethanolamine-N-[methoxy(polyethylene glycol)-550] (18:0 DSPE-PEG550, Mw 1355 g/mol), 1,2-distearoyl-sn-glycero-3-phosphoethanolamine-N-[methoxy(polyethylene glycol)-2000] (DSPE-PEG2000, Mw 2805.5 g/mol), 1,2-Dimyristoyl-rac-glycero-3-methoxypolyethylene glycol-2000 (DMG-PEG2000, Mw 2509.20 g/mol) and 1,2-dioleoyl-3-trimethylammonium-propane (DOTAP, Mw 698.5 g/mol) were purchased by Avanti Polar Lipids (Alabaster, AL, USA). Further, (6Z,9Z,28Z,31Z)-Heptatriaconta-6,9,28,31-tetraen-19-yl 4-(dimethylamino)butanoate (D-Lin-MC3-DMA, 642.1 g/mol) was purchased by BroadPharm (San Diego, CA, USA). Cholesterol (Chol, Mw 386.65 g/mol), L-α-Phosphatidylcholine (eggPC, 95%), Myoglobin (Myo, Mw 17,000 g/mol) from equine heart, Deoxyribonucleic acid, single stranded from salmon testes (ssDNA, 587–831 bp), RNase ZAP™, Dulbecco’s Modified Eagle’s Medium High glucose (DMEM), Dulbecco’s Phosphate Buffered Saline, Foetal bovine serum (FBS), Trypsin-EDTA, Thiazolyl Blue Tetrazolium Bromide (MTT, approx. 98% TLC), Dimethyl sulfoxide (DMSO) and Penicillin–Streptomycin solution were purchased from Sigma-Aldrich (St. Louis, MO, USA). Silencer™ Negative Control siRNA (siRNA, 21 bp) was purchased from Thermo Fisher Scientific (Monza, Italy). The water used was distilled and filtered through 0.22 μm membrane filters (Millipore Corporation, Billerica, MA, USA). Unless specified, all the solvents used were of HPLC grade and all other reagents were of analytical grade.

### 2.2. Microfluidic Production Station for Lipid Nanocarriers

**Figure 1:**
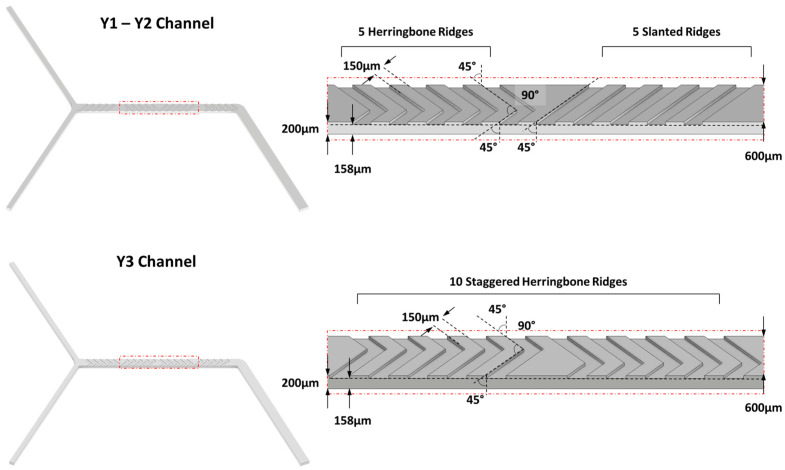
Graphical representation of the different microfluidic chips with details regarding the different geometries of the ridges.

The microfluidic device is in-house assembled, and two syringe pumps (NE-500 OEM, New Era Pump Systems Inc., Farmingdale, NY, USA) are controlled by integrated software created by our laboratory (MKR1010Wifi arduino Microcontroller, Arduino Srl, Monza, Italy controlled by IoT application developed by Blynk v. 2.27.34-Blynk Inc., New York, NY, USA). Syringe pumps can be equipped with syringes of 1–50 mL volume as certificated by the provider. Passive Herringbone Mixer Chip from Microfluidic Chip Shop (Jena, Germany) is made of Cyclic Olefin Polymer (COP) and it presents three Y-shape channels (named Y1, Y2 and Y3 in this work) characterized by a cross-sectional area of 600 × 200 µm but with different micromixer geometry (Figure 1).

Syringes and microchannels are connected by PTFE tubes because of their compatibility with several organic solvents. The software enables the control of the following process parameters: (i) syringes volume, (ii) final formulation volume, (iii) the flow speed into the microchannel (total flow rate—TFR) and (iv) flow rate ratio (FRR) between the two pumps, indicated as aqueous-to-ethanol *v*:*v* ratio. The whole equipment was previously validated by assessing the mixing accuracy, the repeatability and the volume accuracy [17].

### 2.3. Microfluidic Formulation of Liposomes

To set up the manufacturing process of liposomes, ethanol (class III, ICHQ3) was utilized to dissolve neutral lipids such as DSPC and Chol (molar ratio of 77:23). The tested total lipid concentration ranged from 1.1 mM to 3.28 mM. Lipid concentration higher than 3.28 mM was avoided since aggregation phenomena were observed experimentally. Lipid alcoholic solution and phosphate aqueous buffer (10 mM PBS, pH 7.4 were injected into the microfluidic channel (Y-3)) to induce the lipid assembly in the aqueous environment because of the increase of final solution polarity. The production speed was varied from 4 mL/min to 12 mL/min while aqueous-to-ethanol FRRs among 2 and 6 (namely 2:1–6:1 *v*:*v*) were selected. The levels of each operating factor were set based on past reports [18,19,20]. The effect of selected manufacturing parameters on the liposomes’ physical attributes (particle size and polydispersity) was evaluated by a two-level full factorial screening Design of Experiment (DoE). As a subsequent step of optimization, a three-factor Box–Behnken DoE was utilized. DoEs were performed by Statgraphics Centurion 19 software. Process conditions and related numerical values are stated in Table 1.

Table 2 lists the liposomes produced in accordance with the first full factorial screening DoE: 8 samples derived from the combination of the highest and lowest value of the variables and one center point as halfway between the low and high settings (total run = 9). Samples were prepared in triplicate resulting in randomized 21 runs. Since size and PDI were selected as outputs, the liposomes were characterized in terms of size and size uniformity as described in Section 2.5.

Once relevant variables were screened, a second DoE (Box–Behnken design) was used to obtain a surface response and highlight the experimental conditions leading to the optimal response. The Box–Behnken design is a class of surface response design that does not contain an embedded factorial or fractional factorial design. In this design, all factors have three levels: low (−1), center (0) and high (+1). the treatment combinations are at the midpoints of the edges of the process space and the center. Hence, the Box–Behnken design encompassed 15 runs and 3 independent batches for each combination (Table 3).

### 2.4. Effect of the Formulation Variables

The surveyed formulation variables were the organic solvent to dissolve lipid, pH and ionic strength of the aqueous buffer, as well as the lipid composition.

The first aim was to evaluate the effect of the organic solvent selection on the manufacturing process of liposomes. Tested organic solvents, suitable for liposome formation, were ethanol (class III, ICHQ3), methanol (class II, ICHQ3) and isopropanol (class III, ICHQ3). Lipid solution at different concentrations (1.10 mM and 2.19 mM) made of DSPC (77 mol%) and Chol (33 mol%) were used for the mixing at 8 mL/min into Y-3, keeping constant the FRR at 4.

Besides the effect of the organic solvent, the choice of aqueous buffer (25 mM citrate buffer pH 4.5 or 5.5, 10 mM PBS buffer pH 7.4 or 6.8, 10 mM acetate buffer pH 4.5 and 5.5) was investigated. Keeping constant other variables; liposomes were made of DSPC 77 mol% and Chol 33 mol% and prepared at 8 mL/min with a FRR of 4 by using a lipid solution of 1.10 mM.

To point out how liposome manufacturing is influenced by the lipid composition, we compared different DSPC:Chol molar ratios, namely 90:10 mol%, 77:23 mol% and 65:35 mol%. This comparison was carried out by using two total lipid concentrations (1.10 and 2.19 mM) and three FRRs (4, 6 and 9 *v*:*v*).

Finally, we investigated if condensing lipid (DOTAP, 3–10–20 mol%), coating lipids (DSPE-PEG550 and DSPE-PEG2000) as well as structural lipids (DSPC, eggPC) had a role on the nanocarriers physical attributes. The manufacturing process relied on a 1.10 mM lipid solution and 25 mM citrate buffer (pH 5.5) mixed at 8 mL/min. Analysis of liposomes is described in Section 2.5. To streamline the discussion of the experiments, we designate the liposomes generated with DOTAP as cationic liposomes, and those incorporating PEGylated lipids (DSPE-PEG2000 and DSPE-PEG550) as stealth liposomes. Besides the effect of the organic solvent, the choice of aqueous buffer (25 mM citrate buffer pH 4.5 or 5.5, 10 mM PBS buffer pH 7.4 or 6.8, 10 mM acetate buffer pH 4.5 and 5.5) was studied. Other parameters were kept constant; vesicles made of DSPC 77 mol% and Chol 33 mol% were produced at 8 mL/min with a FRR of 4 starting from a lipid solution of 1.10 mM.

#### 2.4.1. Effect of Channels Geometry

With the aim of understanding the effect of the inner channel geometry, liposomes composed of DSPC and Chol at different molar ratios, 77:23 and 65:35, were produced by mixing the lipid solutions and PBS at 8 mL/min into Y-1, Y-2 and Y-3 microchannels. FRRs of 4, 6 and 9 were used. The size and size distribution of obtained liposomes were assessed.

#### 2.4.2. Purification and Buffer Exchange

Y-3 of the Passive Herringbone Micromixer was used for all the optimization studies.

Once the liposomes were prepared, the next step was the solvent removal and the buffer exchange.

First, as soon as the liposomes were produced, the ethanol content was reduced to 10% *v*/*v* by dilution with a proper volume of buffer. After that, lipid-based formulations were dialyzed against 200 mL buffer for 2 h. A comparison between the size and size distribution of liposomes obtained before and after purification was performed.

### 2.5. Nanocarriers Characterization: Size, Surface Charge and Morphology

Dynamic light scattering using a NICOMP 380 ZLS apparatus (Particle Sizing System, Menlo Park, CA, USA) with a 632.8 nm laser and a detection angle of 90° was utilized to evaluate the size and the size distribution of nanocarriers. After dilution in water (dilution factor 2), nanocarriers were analyzed. The water refractive index and viscosity values were 1.330 and 0.887 cP, respectively.

The ζ-potentials were evaluated by electrophoretic light scattering (NICOMP 380 ZLS apparatus, Particle Sizing System, Menlo Park, CA, USA), after the same sample dilution. Results were expressed as mean values ± standard deviation (SD) calculated from at least three independent samples.

The liposome morphology was evaluated by transmission electron microscopy (TEM; JEOL JEM-1200EXIII with TEM CCD camera Mega View III, Tokyo, Japan). Lipid nanocarriers, loaded on a copper grid, were stained with 1% (*w*/*v*) uranyl acetate (negative staining); measurement of NP sizes was carried out by ImageJ software (ImageJ 1.52a, NHI, Bethesda, MD, USA).

### 2.6. Encapsulation Efficiency of Biomolecules

This production station was tested for the feasibility of delivering different macromolecules into lipid nanocarriers. Biomolecules of different natures were selected such as Myo, ssDNA and siRNA. The composition of the lipid nanocarriers was chosen with respect to commercial products.

Myo-loaded NPs were prepared by dissolving Myo into the aqueous buffer before mixing with the lipid solution. The Myo encapsulation into the aqueous core was evaluated by producing liposomes with a similar composition to Onivyde^®^, namely DSPC:Chol:DSPE-PEG550 (lipid solution concentration: 1.10 mM) by testing two different molar ratios (Myo:Lipid) such as 1:20 and 1:10. FFRs of 4 and 6 were tested and hence the initial Myo concentration was modified accordingly (Table 4).

Resulted liposomes were dialyzed against water for 5 h and the unencapsulated protein was detected by UV-vis spectrophotometer at 409 nm according to a Myo calibration curve ranging from 1 to 100 µg/mL.

This study encompassed other biomolecules. siRNA and ssDNA were loaded into LNPs prepared to start from a 1.10 mM lipidic solution made of DOTAP, DSPC, Chol and DSPE PEG2000. An amount of 25 mM citrate buffer (pH 5.5) was used to dissolve oligonucleotides and different N/P (positive amino groups/negative phosphate groups) molar ratios were tested 4 and 6. To test the feasibility of the microfluidic approach for the encapsulation of nucleic acids, LNPs comparable to the benchmark Onpattro^®^ were produced.

SYBR^TM^ Gold assay (Invitrogen - Thermo Fisher Scientific, Monza, Italy) was used to determine the encapsulation efficiency. An aliquot of 100 μL of lipid nanocarrier suspension alone and treated with an equal volume of Triton X were added to 30 μL of SYBR ^TM^ Gold solution. Spectrofluorometer at 485 nm and 535 nm (wavelengths of excitation and emission, respectively) assessed the fluorescence intensity of the samples and the nucleic acids concentration was calculated by a calibration curve obtained from ssDNA and siRNA standard solutions in a concentration rank of 1–100 pmol/mL.

Results are reported as encapsulation efficiency (EE%) defined as the fraction of initial biomolecule amounts that are encapsulated by nanocarriers.

### 2.7. Statistical Analysis

If not otherwise stated, the mean and standard deviation (SD) of at least n = 3 independent batches were calculated to express the results. Statistical significance (*p*-value of less than 0.05) was assessed by one-way or two-way ANOVA tests. GraphPad Prism version 6 (GraphPad Software Inc., La Jolla, CA, USA) and Statgraphics Centurion 19 were used for the statistical analyses.

## 3. Results

Geometry-assisted micromixers are a subset of passive micromixers that rely solely on channel geometry and fluid properties. In a passive mixer, chaotic advection can be created by geometrical modification such as twisted channels, slanted ridges, 90° bends, zigzagging channels, embedded barriers, curved channels and so on [21]. Changing the channel geometry such channel’s curvature and the inner geometry, and the number of staggered/slaved structures generates different secondary flow within the channel thus influencing the mass transfer. Moreover, the height-to-width ratio of the channel cross-section affects the mean velocity [22]. All this evidence suggests that variation in microfluidic devices requires a new optimization of the manufacturing process. Some microfluidic devices have been released on the market and they were used to produce lipid nanocarriers [19,23,24,25]. However, the micromixer used in this work showed some differences and, in the present work, the aim was to investigate the microfluidic manufacturing of different lipid nanocarriers with respect to the most famous liposomal nanoformulations available on the market. Generally, lipid carriers were produced in microfluidic device by ethanol-dilution method. Vesicles formation follows four steps: aggregation of the lipid moieties, formation of the intermediate disk-like structure, fusion of these intermediate disk-like structures and transformation of disk-like structures to close vesicles.

At first instance, glycerophospholipids and Chol were selected since they are the basic components of the market products. Among glycerophospholipids, DSPC was used because it is present in several liposomal formulations, and it is a neutral synthetic lipid with a well-defined composition and purity. The effect of lipid concentration and microfluidic parameters (TFR and FRR) were assessed by exploiting a two-level full factorial screening DoE; the statical approach based on DoE has been investigated in different studies and the usefulness has been demonstrated in the development of lipid-based nanoformulation [26,27,28]. For DoE, a common lipid composition of 77 mol% DSPC and 23 mol% Chol was selected by results from the literature and by selected benchmark DaunoXome^®^ [29,30]. DoE experiments and relative results are reported in Table 5. As it can be noted, the first variation from the other microfluidic devices is the total lipid concentration that was decreased below 3.28 mM to avoid channel clogging. This phenomenon can be related to the different cross-sectional areas of the channel and the subsequent change in the fluid pressure in the channel.

**Figure 2:**
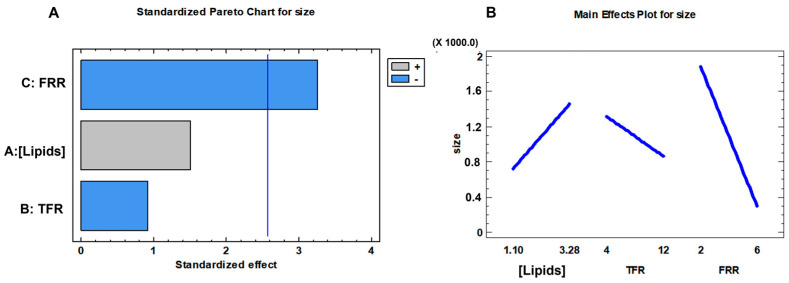
Screening DoE data elaboration for liposome size: (**A**) Pareto chart and (**B**) main effect plot.

Elaboration of data in Table 5 was performed by Statgraphics Centurion 19 software to point out the production conditions affecting the size of neutral liposomes. As shown in Figure 2A, FRR is the main parameter influencing the mean size of liposomes (*p* value = 0.0224). Significant improvements in nanocarrier physical features were observed by increasing the FRR from 2 (DoE 9: ~1700 nm, PDI = 0.76) to 6 (DoE 4: 222 nm, PDI = 0.29).

The findings align with the concept underlying liposome formation: upon rapid dilution of ethanol with a buffer to a critical concentration of 60–80%, small-sized vesicles are generated [31].

Instead, a slow ethanol dilution triggers large-sized vesicles. The effect of each parameter on the liposome size is shown in Figure 2B. An enlargement of the carrier was revealed because of an increase in the lipid concentration. Sample DoE 1 and sample DoE 3 (Table 5) changed the mean diameter from 877 ± 20 nm to 224 ± 10 nm by reducing the lipids concentration from 3.28 mM to 1.10 mM, keeping the TFR and FRR constant (4 mL/min and 6, respectively). An increase in the lipid concentration changes the solvent fluid viscosity triggering an alteration of the mixing efficiency that is not negligible. The same behavior was spotted for samples DoE 8 and DoE 4 (Table 5) when 12 mL/min TFR and 6 FRR were set. As regards the TFR, Figure 2B points out that high TFRs downsize the liposomes. The dimensions of sample DoE 1 and sample DoE 8 were decreased from 877 ± 20 nm to 266 ± 2 nm by increasing the production speed from 4 to 12 mL/min.

To identify the best optimal operative conditions through a 3D surface representation, a Box–Behnken DoE was run to achieve a second-order model. The three-factor Box–Behnken design is almost rotatable, meaning that all the design points are at the same distance from the center of the design which leads itself to aptly create a response surface plot. The results in terms of size and PDI were stated in Table 6, while the descriptive statistics of the Box–Behnken test design are in Table S1 (Supplementary Materials).

The regression equation, found from the experimental data by Statgraphics Centurion, is as follows:

Liposomes mean size = 2550.9 + 469.8 × [lipids] − 195.1 × TFR – 758.9 × FRR − 47.6 × [lipids] × TFR + 16.7 × TFR^2^ + 67.0 × FRR^2^.

The accuracy of the multiple regression equations was high. The R^2^ of 86.4% was revealed for particle size.

**Figure 3:**
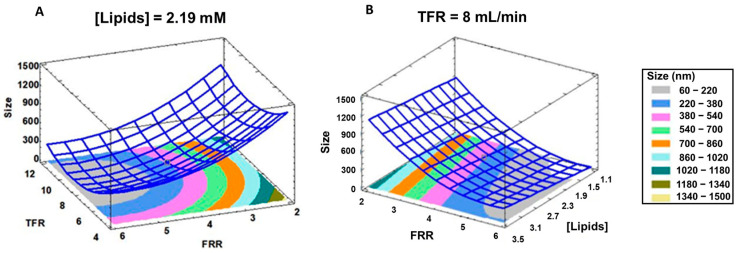
Response surface plot for neutral liposome size. (**A**) Predicted size in relation to TFR and FRR, lipids concentration was constant at 2.19 mM. (**B**) Predicted size in relation to FFR and lipids concentration [Lipids]. TFR was 8 mL/min.

Figure 3 depicts the three-dimensional graphic representation of this equation.

The experimental design space defined by TFR and FRR to obtain liposomes in a desired dimensional range by using a total lipid concentration of 2.19 mM is displayed in Figure 3. As can be seen, it is recommended to work at TFR higher than 5 mL/min with a FRR ranging from 4.5 to 6 to gather liposomes with a mean size lower than 400 nm (blue and grey area, Figure 3A). Likewise, Figure 3B shows the liposome variation as a function of the lipid concentration and the FRR setting the production speed at 8 mL/min. In this case, by using the lowest lipid concentration (1.10 mM) and a FRR higher than 4.5, liposomes ranging from 60 nm to 220 nm can be produced. These liposomes are like the benchmark DaunoXome^®^ and are compatible with intravenous administration.

As known for the literature, the homogeneity can be improved by reducing the ethanol concentration in the sample through dilution or buffer exchange [19,32]. As a general remark about the size distribution, the PDIs reported in Table 5 and Table 6 (samples without purification) ranged from 0.21 ± 0.11 to 0.93 ± 0.1 for samples DoE 2 and DoE 5, respectively. The next studies (Section 3.2) figure out the effect of the purification.

### 3.1. Effect of the Formulation Parameters

The further goal was to assess the formulation variables. At first instance, the focus was on the choice of the organic solvent used for the lipid dissolution. Among the organic solvents, methanol and ethanol as well as isopropanol are water miscible and all of them can be used in microfluidic manufacturing of lipid nanocarriers.

**Figure 4:**
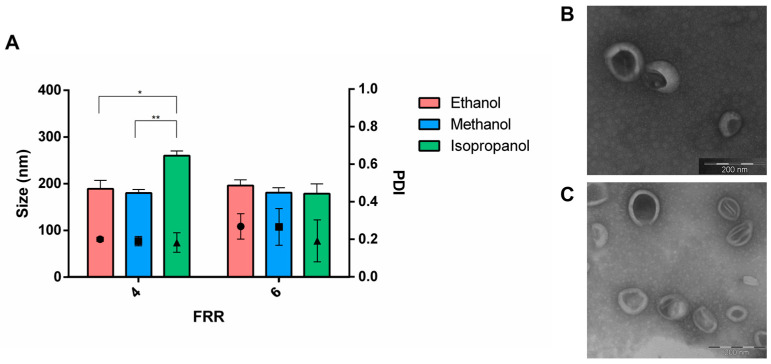
(**A**) Effect of the organic solvent on the liposome formation: produced vesicles were characterized in terms of size and PDI. Ethanol (red bar), methanol (blue bar) and isopropanol (green bar) were used. Sidak’s multiple comparison tests revealed statistical significance for * *p*-value < 0.05 and ** *p*-value < 0.01. (**B**) TEM image of liposomes produced by using ethanol and FRR of 4. (**C**) TEM image of liposomes produced by using ethanol and FRR of 6. Scale bar of 200 nm.

As reported in the literature, it is important to note that water miscibility of the organic solvent is not the sole factor to consider. It is clear that also the polarity of the organic solvent can influence both lipid solubility and the self-assembly process [33]. The size and the size distribution of liposomes (DSPC:Chol 77:23 mol%), produced by using methanol, ethanol and isopropanol as solvent at TFR of 8 mL/min and FRR of 4, are shown in Figure 4A.

A slight impact of the solvent on the size was pointed out. Larger vesicles were obtained as the polarity decreased: liposomes increased in size from 180 nm to 195 nm by using methanol and ethanol, respectively. Upon switching to isopropanol, the size of the liposomes increased to up to 260 nm. Across the formulations, PDI was in the range of 0.2 and 0.4, regardless of the organic solvent selected. When the FRR was increased to 6, vesicles remained similar in size (approximately 180 nm), with no notable impact from the solvent used for manufacturing. The general trend of vesicle size as a function of the organic solvent observed in this work, namely isopropanol > ethanol > methanol was in accordance with data from the literature collected for other commercially available microfluidic devices [25,31]. Moreover, Webb et al. (2019) demonstrated that a key consideration during lipid nanocarrier manufacture is the choice of the solvent since the dimension of the nanocarriers was reduced by increasing the polarity of the organic solvent or organic solvent mixture [31]. Moreover, Roces et al. (2020) proved that the effect of the solvent depended on the formulation composition since the addition of PEG to liposomes consisting of DSPC:Chol undo the solvent effect [25]. According to the results, both methanol and ethanol are suitable for the microfluidic manufacturing of lipid nanocarriers. However, considering safety concerns, ethanol may be the preferred option. About the microfluidic technology, the device here proposed is comparable to commercial platforms. Spherical vesicular structures with a diameter of around 150 nm were revealed by TEM. No evident differences were observed by using FRR 4 (Figure 4B) and 6 (Figure 4C) when lipids were dissolved in ethanol.

**Figure 5:**
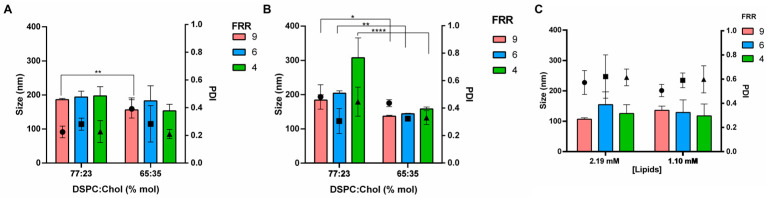
Effect of formulation variables on liposome size and size distribution. (**A**) Effect of the Chol concentration by using a lipid concentration of 1.1 mM and 8 mL/min TFR, three FRRs were tested: 9 (red bar), 6 (blue bar) and 4 (green bar). ** *p*-value < 0.01. (**B**) Effect of the Chol concentration by using a lipid concentration of 2.19 mM and 8 mL/min TFR, 3 FRRs were tested: 9 (red bar), 6 (blue bar) and 4 (green bar). * *p*-value < 0.05, ** *p*-value < 0.01, **** *p*-value < 0.0001. (**C**) Impact of eggPC as structural lipid (eggPC:Chol 77:23 mol%); 2 lipid concentrations were tested: 1.10 and 2.19 mM.

Next, we sought out how the composition influenced the particle size and PDI of the liposomal formulation. Initially, we examined the impact of varying Chol concentrations. Chol’s primary characteristic is its capacity to regulate the rigidity and permeability of lipid membranes. Consequently, its concentration in the liposomal formulation was carefully adjusted to fine-tune both payload release and formulation stability. The Chol content is a key factor since it influences the transition temperature (Tc) of liposome bilayers. Without Chol, the lipid hydrocarbon chain into the bilayer crystalized in the rigid crystalline phase at the Tc of the pure phospholipid (i.e., 55 °C for DSPC). When the Chol concentration rises to 33 mol% the Tc is no longer detectable [34]. For that reason, several DSPC:Chol molar ratios were screened, namely 90:10–77:23–65:35 mol%. Figure 5 shows the results obtained by incrementally increasing the Chol molar concentration, consequently reducing the molar percentage of neutral lipid (DSPC), while keeping the lipid concentration constant. At the lowest Chol concentration (10 mol%), acceptable results were obtained only by the highest FRR tested (9 *v*/*v*) and the lowest lipids concentration (1.10 mM) when liposomes of 286 ± 27 nm were observed. On the other hand, a broad particle size distribution with particles greater than 1 μm was revealed by using lower FRR of 4 and 6 *v*/*v*. As a means of the increased Chol concentration (23–35 mol%), vesicles achieved a diameter lower than 200 nm (Figure 5A). With the exception of FRR 9, liposomes with Chol concentrations of 23 mol% and 35 mol% showed no notable size variations across the range of FRRs tested.

By using a final lipid concentration of 2.19 mM (Figure 5B), the physical quality attributes of the vesicles were affected by the lipid composition, and the increase of the Chol molar percentage from 23 mol% to 35 mol% notably downsized the liposomes for all the FRRs tested. This result can be ascribed to the increase of the lipid membrane elasticity. The vesicles’ mean diameter varied from 184 ± 15 nm to 114 ± 6 for the FRR of 9 (*p* value < 0.05, Sidak’s multiple comparison test), from 204 ± 7 nm to 155 ± 9 nm for the FRR of 6 (*p* value < 0.05, Sidak’s multiple comparison test) and, finally, from 308± 58 nm a 154 ± 3 nm for FRR of 4 (*p* value < 0.05, Sidak’s multiple comparison test). Moreover, the highest Chol concentration (35 mol%) negates the influence of the FRR on liposome size, as mean diameters consistently remained below 160 nm for all FRRs tested. As previously observed, enhancing the Chol concentration in the formulation led to the formation of the smallest vesicles. Across the formulations, PDIs ranged from 0.2 and 0.4 and the Chol molar concentration did not affect this attribute. Once the effect of the Chol on liposome manufacturing was evaluated, the structural lipid (DSPC) was changed by eggPC, a mixture of phosphatidylcholine with fatty acids of different carbon chain lengths derived from natural source, used for the preparation of the commercial product Myocet^®^ (Multi-lamellar Vesicles with sizes of 80–90 nm). Alcoholic solutions of egg PC and Chol (77:23 mol%) at two different concentrations (2.19 mM and 1.10 mM) were mixed into the microfluidic channel at different FRRs of 4, 6 and 9. Produced liposomes were heterogeneous in size (PDI > 0.4) with a mean diameter smaller than 160 nm (Figure 5C). The heterogeneity can be ascribed to the unknown composition of eggPC.

**Figure 6:**
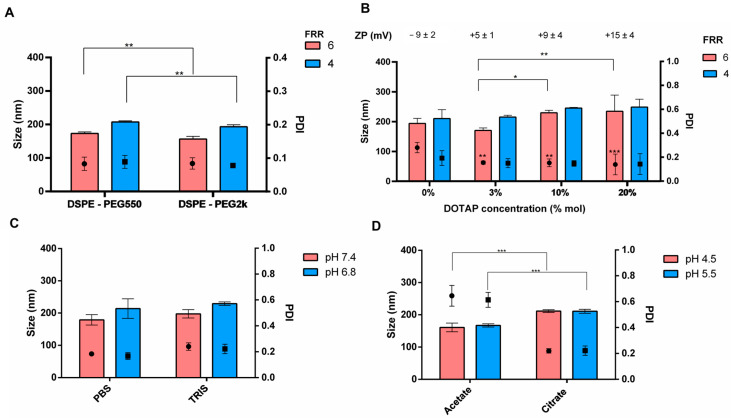
Effect of formulation variables on liposomes size and size distribution: (**A**) Production of nanocarrier made of DSPC, Chol, PEGylated lipid (DSPE-PEG550 or DSPE-PEG550) at the molar ratio 77:20:3 mol%. Lipid concentration was 1.1 mL and stealth liposomes were prepared at 8 mL/min TFR and 6 (red bar) or 4 (blue bar) FRR. (**B**) Production of cationic liposomes made of DSPC, Chol and DOTAP (3%, 10% and 20 mol%) starting for a lipid solution of 1.10 mM. Operating parameters: TFR of 8 mL/min and FRR of 6 (red bar) or 4 (blue bar). (**C**) Impact of the aqueous buffer on liposomes (77:23 mol% DSPC:Chol). Operating parameters: TFR of 8 mL/min and FRR of 4. (**D**) Impact of the aqueous buffer on the production of cationic liposomes (77:20:3 mol% DSPC:Chol: DOTAP). Operating parameters: TFR of 8 mL/min and FRR of 6. *, **, *** mean *p*-value < 0.05, <0.001, <0.0001, respectively.

To complete the investigation of the influence of the lipid composition on the microfluidic manufacturing process and the feasibility of the new platform to produce a wide range of liposomes, PEGylated liposomes and cationic liposomes were produced. For the first one, DSPE-PEG550 or DSPE-PEG2000 was added to the lipid solution, and the molar ratio DSPC:Chol:DSPE-PEG of 77:20:3 mol% was tested (Figure 6A) using Onivyde^®^ as a reference. The inclusion of PEG-lipid has a slight impact on the nanocarriers’ size; however, it notably enhances homogeneity, with PDIs consistently below 0.1. More in detail, PDIs were 0.08 ± 0.02 and 0.09 ± 0.02 for FRR of 6 and 4, respectively, by using DSPE-PEG550 and 0.09 ± 0.02 and 0.08 ± 0.01 for FRR of 6 and 4, respectively, when DSPE-PEG2000 was used. Regarding the liposome size, collected data confirmed the previous observations regarding the influence of the FFR. Furthermore, the incorporation of DSPE-PEG2k into the bilayer led to smaller carriers: they had sizes of 157 ± 4 nm and 193 ± 3 nm for a FRR of 6 and 4, respectively; noticeable differences were observed when compared to vesicles containing DSPE-PEG550 (173 ± 2 nm and 207 ± 2 nm for FRR 6 and 4). Ultimately, in light of the growing demand for charged liposomes, particularly for delivering negatively charged biomolecules like siRNA and mRNA, cationic liposomes were synthesized by using increasing concentrations of DOTAP (3, 10 and 20 mol%), keeping the total lipid concentration constant at 1.10 mM. The results are stated in Figure 6B. The size increased as long as the DOTAP concentration increased, diameters of 171 ± 8 nm and 230 ± 8 nm were revealed by changing the DOTAP% from 3% to 10 mol% (* *p*-value = 0.036—Tukey’s multiple comparison test). Similar behavior was highlighted by using a DOTAP concentration of 20 mol% (235 ± 54 nm; ** *p*-value di 0.006—Tukey’s multiple comparison test). Since the total lipid concentration was constant the enlargement was ascribed to the cationic lipid. The addition of DOTAP to the neutral liposomal formulation significantly improved the homogeneity of the liposome population. PDI values were always lower than 0.2 when DOTAP was present. As expected, the charge of the lipid bilayer underwent inversion due to the presence of the cationic lipid; the surface charge of DSPC:Chol-based liposomes was 9 ± 2 mV, whilst the positive surface charge of 5 ± 1 mV, +9 ± 3 mV and +15 ± 4 mV were detected for cationic liposomes with 3%, 10% and 20 mol% DOTAP, respectively.

In addition to lipid content, another crucial variable to consider is the aqueous buffer. Therefore, the selection of the aqueous buffer was thoroughly investigated. Ionic strength and pH are key parameters during lipid nanocarriers manufacturing. Low pH aqueous buffers are usually used for delivering nucleic acids instead neutral pH aqueous buffers are used for the preparation of liposomes for protein delivery [18,31,35]. Therefore, it was examined the effect of PBS and TRIS buffer as neutral aqueous media (6.8–7.4 pH) on neutral liposomes (DSPC/Chol) (Figure 6C). As shown in Figure 6C, a decrease of the buffer pH from 7.4 to 6.8 caused a slight increase in the average size from 180 nm to 213 nm and from 198 nm and 229 nm when PBS and TRIS were used, respectively; meanwhile, the size distribution was not affected. No significant differences in dimensions were pointed out by PBS and TRIS buffers. By these means, we can conclude that both PBS and TRIS buffers are useful media to produce neutral liposomes. Figure 6D likewise shows features of cationic liposomes (DOTAP 10 mol%) prepared at pH 4.5 (red bars, Figure 6D) and pH 5.5 (blue bars, Figure 6D) through citrate and acetate buffers, whilst the ionic strength was preserved at 25 mM. Acetate buffer allowed us to obtain smaller but unstable cationic liposomes with a mean diameter of 170 nm and, still, this vesicle population was also characterized by a notable heterogenicity (PDI > 0.4); conversely, citrate buffer permitted us to produce larger homogenous liposomes with a size around 200 nm and an acceptable PDI of 0.2. Indeed, from this experimental setting, it can be assumed that the quality attributes of cationic liposomes can be affected by the composition of aqueous buffer, and it was hypothesized that specific electrolytes may alter the lipid head repulsion, thus affecting the lipids package into the bilayer. Therefore, citrate buffer should be preferred to produce cationic liposomes through the microfluidic platform developed in this study; moreover, it was reported in the literature [19] that if the payload is an RNA moiety, the citrate buffer is able to reduce the RNA hydrolysis by the virtue of the chelating effect of sodium citrate.

### 3.2. Effect of the Channel Geometry

**Figure 7:**
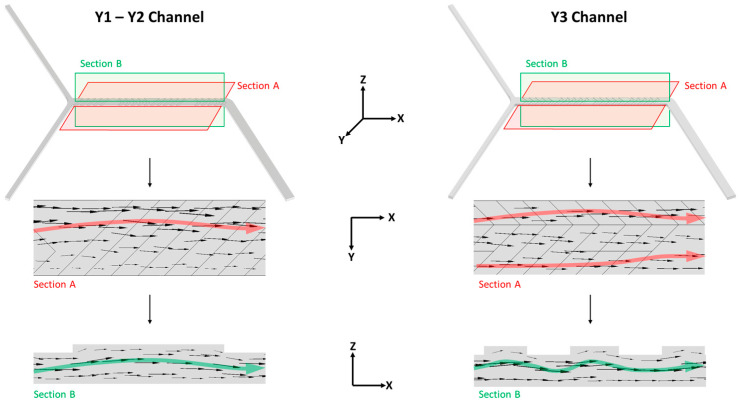
CAD design of the two different micromixers adopted in this study (first row); visualization of the tangential projection of the velocity magnitude vectors onto the plane defined by Section A (second row); visualization of the tangential projection of the velocity magnitude onto the plane defined by Section B (third row).

**Figure 8:**
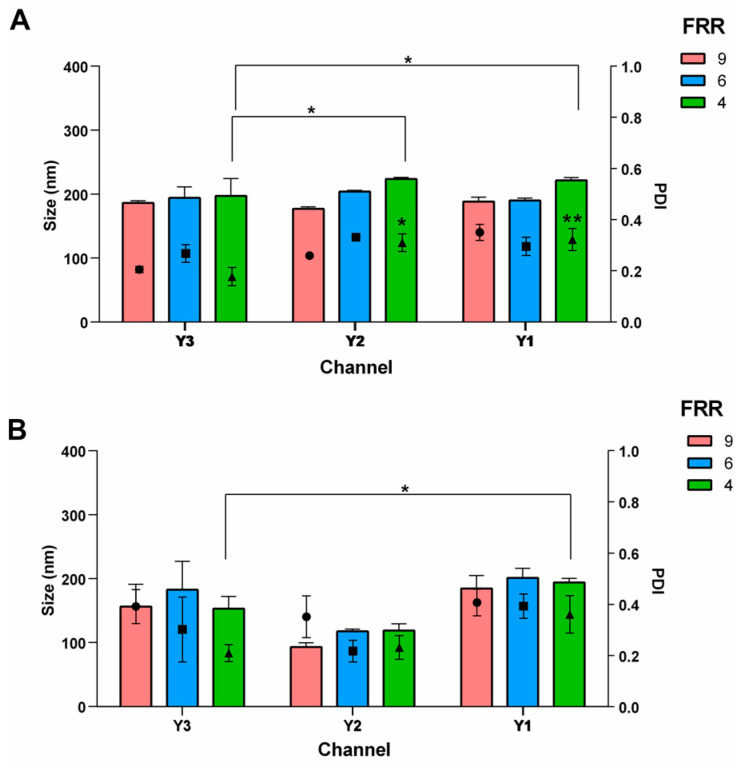
Effect of the channel geometry on the liposomes physical features of (**A**) liposomes made of DSPC and Chol at the molar ratio of 77:23 mol% and (**B**) liposomes made of DSPC and Chol at the molar ratio of 65:35 mol%. FRR was changed to 9 (red bar), 6 (blue bar) and 4 (green bar), while lipid concentration and TFR were kept constant, namely 1.1 mM and 8 mL/min. ANOVA test revealed differences for * *p*-value < 0.05 and ** *p*-value < 0.01.

The influence of channel geometry (Y1, Y2, Y3—Figure 7) was evaluated using a lipid mixture comprising DSPC and Chol (77:23 and 65:35 mol%) at a lipid concentration of 1.10 mM. The microfluidic manufacturing varied the FRR while maintaining a constant TFR of 8 mL/min. Figure 8A depicts the results of liposomes formulated with 77:23 mol% DSPC and Chol. Statistical analysis did not reveal any difference among the microchannels except for liposomes produced by FRR 4 when an increase in size was noted by using Channel Y1 and Y2 (*p* value of 0.0312 and 0.0170, respectively, if compared to Y3). In the Y-3 channel, the presence of a staggered herringbone structure leads to a more chaotic flow regime due to the presence of a second high-velocity region that is not present in slanted ridge structures (Figure 7).

The channel architecture may have played a significant role when employing a low FRR. Furthermore, utilizing Y1 and Y2 combined with a low FRR (4) increased the heterogeneity of the batches. PDIs were 0.18, 0.31 and 0.32 for Y3, Y2 and Y1, respectively. Figure 8B shows the effect of the channel geometry on the liposomes based on 65:35 mol% DSPC:Chol, which shows Y2 led to the production of smaller vesicles (size < 120 nm); however, it should be noted that the intensity signal from these samples was notably low, suggesting a low concentration of liposomes. As for the previous formulation, Y1 and Y3 did not affect the physical features of the highest FRR 9 and 6 (red and blue bars, respectively), while enlargement of the vesicles was detected when liposomes were produced by Y1 at the lowest FRR (4, green bar).

### 3.3. Purification and Buffer Exchange of Liposomes

While evaluating the feasibility of a newly assembled microfluidic platform for producing lipid-based nanocarriers, we also performed an assessment of the purification process. The aim was to eliminate the alcoholic solvent to reach ethanol levels that meet clinical standards for acceptability. This involved examining a wide range of operating parameters to optimize production and implementing purification methods tailored to remove ethanol while ensuring the integrity, stability and functionality of the nanocarriers [36]. During the development of nanosized drug delivery systems, the purification step is a notable hurdle and dialysis is commonly used as a purification method in the early stage of development.

**Figure 9:**
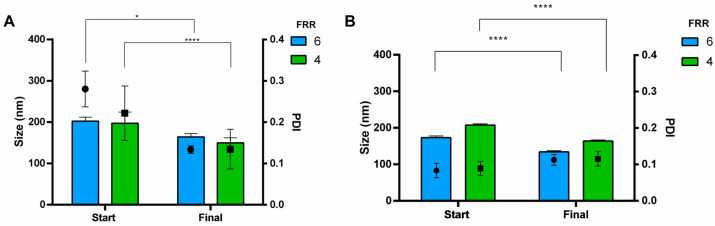
Liposome size and size distribution before (start) and after (final) dialysis of (**A**) neutral liposomes (DSPC:Chol 77:23 mol%) and (**B**) stealth liposomes (3 mol% DSPE PEG550). FRR was set at 6 (blue bar) and 4 (green bar), while lipid concentration and TFR were kept constant, namely 1.1 mM and 8 mL/min. ANOVA test revealed differences for * *p*-value < 0.05 and **** *p*-value < 0.0001.

To explore the impact of buffer exchange on liposomal production via microfluidics, liposomes generated through microfluidic methods were subjected to dialysis against PB at a pH of 7.4. Results indicated that simply diluting to reduce alcohol concentration is insufficient, underlining the necessity of buffer exchange to enhance the quality attributes of the product, so technology improvement is needed in the view of continuous flow manufacturing. After dialysis, size was significantly reduced for all the formulations tested. For neutral liposomes, stated in Figure 9A, size was reduced from 202 to 159 nm (FRR of 6) and from 197 to 150 nm (FRR of 4). Moreover, all the PDIs were below 0.2. Purification slightly increased the stealth liposome size to 134 ± 3 nm and 164.0 ± 2.8 nm for FRR 6 and 4, respectively (Figure 9B). Likewise, stealth liposomes were homogeneous in size with PDI of 0.113 ± 0.02 and of 0.115 ± 0.02 for FRR 6 and 4.

**Figure 10:**
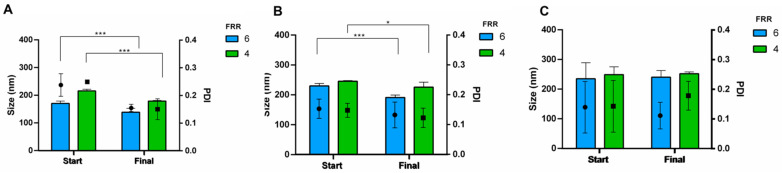
Size and size distribution before (start) and after (final) dialysis of cationic liposomes with different concentrations of DOTAP: (**A**) 3 mol%, (**B**) 10 mol% and (**C**) 20 mol%. FRR was set at 6 (blue bar) and 4 (green bar), while lipid concentration and TFR were kept constant, namely 1.1 mM and 8 mL/min. ANOVA test revealed differences for * *p*-value < 0.05 and *** *p*-value < 0.001.

Similar behaviour was observed for cationic liposomes as reported in Figure 10. By removing the alcohol residues, liposomes (3 mol% DOTAP) showed size in the rank of 150—200 nm with PDIs below 0.2 (Figure 10A). A notable reduction in particle size (*p*-value < 0.05, Sidak’s multiple comparison test) was assessed with 10 mol% DOTAP, while PDIs were invariant below 0.2 (Figure 10B). Liposomes mean size of 252 nm (PDI = 0.178) and 240 nm (PDI = 0.111) were assessed by DOTAP 20% after purification for FRR 4 and 6, respectively (Figure 10C). It can be noted from Figure 10C that no statistical differences were highlighted across the formulation; however, a reduction of standard deviation between batches was observed after the purification.

### 3.4. Encapsulation Efficiency of Biomolecules

**Figure 11:**
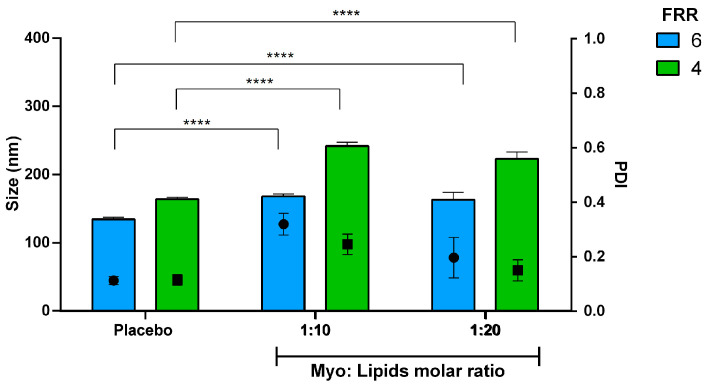
Effect of Myo encapsulation into stealth liposomes (DSPC:Chol:DSPE-PEG550 77:20:3 mol%): size and size distribution. FRR was set at 6 (blue bar) and 4 (green bar) while lipid concentration and TFR were kept constant, namely 1.10 mM and 8 mL/min. ANOVA test revealed differences for **** *p*-value < 0.0001.

The microfluidic system was utilized for the manufacture of lipid nanocarriers loaded with various biomolecules. This study centered on the delivery of biomolecules, such as proteins and oligonucleotides, as they are key tools in healthcare. However, despite their importance, there remain several limitations associated with their delivery. First, Myoglobin (Myo) as a model protein of 17 kDa was loaded into stealth liposomes made of DSPC, Chol and DSPE-PEG550. Two different Myo-to-lipids molar ratios were tested, namely 1:10 and 1:20. As can be seen from Figure 11, at the same FRR, an enlargement of the liposomes was triggered by Myo encapsulation for both the molar ratios, and this evidence confirmed the presence of the macromolecules in the inner core of nanosystems; moreover, we did not point out any differences between the two different drug amounts.

By using FRR 6, stealth liposomes showed sizes of 168 ± 3 nm and 163 ± 11 nm with a Myo-to-lipids molar ratio of 1:10 and 1:20, respectively. Otherwise, larger vesicles were collected by FRR of 4, showing mean dimensions of 242 ± 6 nm and 223 ± 10 nm for Myo-to-lipids molar ratio of 1:10 and 1:20, respectively. As expected, encapsulating macromolecules also increased the heterogeneity of the vesicle population.

Effective encapsulation of Myo was attained across the formulations; it ranged from 72% to 79% for all the microfluidic settings tested. Keeping the Myo-to-lipids molar ratio constant at 1:10 the calculated encapsulation efficiencies were 72.6 ± 0.6% for FRR of 6 and 74.5 ± 0.3% for FRR of 4. Instead, by using a 1:20 Myo-to-lipids molar ratio, a slight but not significant increase of the Myo encapsulation was revealed (78.8 ± 1.0% and 75.7 ± 0.9% for FRR of 6 and 4, respectively).

The next step involved assessing the influence of encapsulating nucleic acids within these nanosystems. Therapeutic nucleic acids serve various functions such as gene inhibition, editing, repair or replacement, and encompass a range of weights, geometries and compositions. In this study, model siRNA (21 bp) and ssDNA (587–831 bp) were chosen. Therefore, LNPs were prepared at 4 FRR and 8 mL/min TFR by dissolving the nucleic acids in the aqueous buffer (25 mM citrate pH 5.5). Both the nucleic acids were loaded at an N/P ratio of 4 and 8. Similar to Myo, the entrapment of nucleic acids led to an increase in the size of the LNPs. Concerning siRNA as payload, homogenous LNPs were obtained with a size of 392 ± 22 nm (PDI = 0.10 ± 0.01) for a N/P ratio of 4 and 372 ± 7 nm (PDI = 0.08 ± 0.01) for a N/P ratio of 8. A high encapsulation efficiency of around 96% was achieved for all the siRNA-loaded LNPs corresponding to an entrapped siRNA concentration of 570 pmol/mL and 282 pmol/mL. For ssDNA, by using N/P of 4, LNPs were 384 ± 10 nm in size (PDI = 0.109 ± 0.031) with a mean encapsulation efficiency of 75% (26.4 pmol/mL), whereas the highest NP ratio leads to LNPs of 324 ± 16 nm and an encapsulation efficiency of the 81% (siRNA concentration of 17.8 pmol/mL). Additionally, these findings demonstrated an improved size distribution of the loaded LNPs, indicating that encapsulating nucleic acids into LNPs enhances particle stability through electrostatic interactions between the positively charged lipid and the negatively charged payload. The biomolecule encapsulation efficiencies achieved by the newly assembled microfluidic device in this study were consistent with those of other microfluidic equipment available on the market. Definitely, the cargo caused an important enlargement of LNPs that are suitable for a local injection; however, they become inconsistent for an intravenous administration. For this reason, even if we figured out the skills of the microfluidic platform, further characterization of the formulation variables is needed according to the specific application. Within this work, to assess the feasibility of the new apparatus, the benchmark Onpattro^®^ was successfully replicated. LNPs made of D-Lin-MC3-DMA, Chol, DSPC and DMG-PEG showed a size of 154 ± 26 nm (PDI = 0.1 ± 0.1) and an entrapped siRNA concentration of 320 pmol/mL. Indeed, the process parameters set in this work allow us to obtain LNPs with physic attributes comparable to commercial products and consistent with an intravenous injection.

## 4. Discussion

Lipid nanocarriers are considered breakthrough technology for drug delivery, mainly for biomolecules such as proteins and nucleic acids. Therefore, the development of production technologies for biomolecules-loaded lipid nanocarriers with high reproducibility is strongly desired. Over the past few years, micromixing has become a major topic of research interest because of the advancement in microfluidic applications [37]. The performance of any micromixer is related to the homogenous and rapid mixing of species that is influenced by channel geometry [38]. Here, we tested a new in-house developed microfluidic platform to produce lipid nanocarriers, using micromixers with staggered/slanted structures. Even if the channel geometry seems to be similar to other microchannels mounted on branded microfluidics platforms, there are some differences such as the depth ratio of the groove, the asymmetry index, groove intersection angle, upstream-to-downstream channel width ratio, groove number, half-cycle number and, overall, the cross-section area of the channel. All these features influence the mixing efficiency of the device, and the manufacturing process needs to be optimized if one of these geometrical features is changed. First, the difference with respect to the commercial device is the working total lipid concentration that must be below 3.28 mM to avoid channel clogging. This is mainly due to the larger cross-sectional area of the channel and lower inner fluid flow. The process was set up by a DoE to systematically evaluate the process parameters. Flow rate ratio and lipid concentration are the main parameters to be tuned for downsizing the lipidic nanocarrier because of the rapid dilution of lipidic alcoholic solution into the aqueous buffer. To implement the DoE approach different liposomes were prepared namely neutral, stealth and cationic liposomes. For the organic solvent used to prepare the lipidic working solution, larger vesicles were obtained by decreasing the polarity of the organic solvent. A general trend was observed in isopropanol > ethanol > methanol. The polarity of the solvent influenced the kinetics of the lipid assembly into vesicles regardless of the microchannel geometry; indeed, this trend agreed with another microfluidic device. Materials were chosen based on the most famous liposomal formulations on the market. With regards to the type of lipids, the use of a natural mixture of phosphatidylcholine (e.g., from egg and soy) allowed for increasing the vesicles’ heterogenicity because unsaturated phospholipids (such as the egg or soybean phosphatidylcholine) provide less-stable bilayer, because of the disturbance of the packing effect of close acyl chains and exhibit higher flexibility. PEGylated phospholipids help to stabilize the vesicles since the presence of PEG chains on the surface reduces the energy barrier resulting from the balance between attractive and repulsive forces; thus, the aggregation of two nanocarriers while approaching one another is prevented. The addition of DOTAP increases the repulsive force between the lamellae and thus leads to vesicle enlargement. This work also demonstrated the feasibility of the encapsulation of biomolecules. Good results were obtained for the loading of a model protein (Myoglobin) into stealth liposomes. On the contrary, the nucleic acids caused an increase in the LNP sizes that are inconsistent for an intravenous administration suggesting that the formulation needs to be optimized according to the application. In this context, the Onpattro**^®^** formulation was successfully replicated.

## 5. Conclusions

To date, lipid nanocarriers are leveraged to deliver biomolecules, mainly nucleic acids and proteins, yet the complete realization of the potential of liposomes remains a significant concern. Winning bench-to-bedside application has been limited due to persistent process-related challenges in manufacturing like batch-to-batch reproducibility, low drug entrapment, time-consuming problems and, overall, scale-up problems. Here, we have introduced a microfluidic-assisted liposome manufacturing approach that offers a promising alternative to conventional microfluidic devices, addressing these issues and potentially facilitating smoother translation into practical applications. We demonstrated that geometrical differences of the microfluidic cartridge notably affect lipid assembly in the microchannel and thus the manufacturing process. Hence, we provide a useful guide to help the optimization of lipid-based nanocarrier manufacturing.

## Figures and Tables

**Table 1:** Selected factors and relative level for DoE study.

Factors		Level		
	Category	−1	0	+1
Lipid concentration	Continuous	1.10 mM	2.19 mM	3.28 mM
Total flow rate, TFR	Continuous	4 mL/min	8 mL/min	12 mL/min
Aqueous-to-ethanol flow rate ratio, FRR	Continuous	2	4	6

**Table 2:** Selected factors and relative level for full factorial screening Design of Experiment.

Batch Code	Lipid Concentration (mM)	TFR (mL/min)	FRR (*v:v*)
DoE 1	3.28 (+1)	4 (−1)	6 (+1)
DoE 2	3.28 (+1)	4 (−1)	2 (−1)
DoE 3	1.10 (−1)	4 (−1)	6 (+1)
DoE 4	1.10 (−1)	12 (+1)	6 (+1)
DoE 5	3.28 (+1)	12 (+1)	2 (−1)
DoE 6	1.10 (−1)	4 (−1)	2 (−1)
DoE 7	2.19 (0)	8 (0)	4 (0)
DoE 8	3.28 (+1)	12 (+1)	6 (+1)
DoE 9	1.10 (−1)	12 (+1)	2 (−1)

**Table 3:** Selected factors and relative level for Box–Behnken design.

Batch Code	Lipid Concentration (mM)	TFR (mL/min)	FRR (*v*:*v*)
DoE 10	3.28 (+1)	8 (0)	2 (−1)
DoE 11	2.19 (0)	12 (+1)	6 (+1)
DoE 12	3.28 (+1)	4 (−1)	4 (0)
DoE 13	3.28 (+1)	12 (+1)	4 (0)
DoE 14	2.19 (0)	8 (0)	4 (0)
DoE 15	2.19 (0)	4 (−1)	6 (+1)
DoE 16	1.10 (−1)	8 (0)	2 (−1)
DoE 17	1.10 (−1)	8 (0)	6 (+1)
DoE 18	2.19 (0)	4 (−1)	2 (−1)
DoE 19	3.28 (+1)	8 (0)	6 (+1)
DoE 20	1.10 (−1)	4 (−1)	4 (0)
DoE 21	2.19 (0)	8 (0)	4 (0)
DoE 22	2.19 (0)	12 (+1)	2 (−1)
DoE 23	1.10 (−1)	12 (+1)	4 (0)
DoE 24	2.19 (0)	8 (0)	4 (0)

**Table 4:** Myo concentration in the aqueous buffer.

Molar Ratio Myo:Lipids	FRR 4	FRR 6
1:10	0.323 mg/mL	0.484 mg/mL
1:20	0.162 mg/mL	0.242 mg/mL

**Table 5:** DoE screening design results: neutral liposome size and PDI (n = 3 independent experiments). The statistical significance is reported by the *p*-value of three different ANOVA tests.

Batch Code	[Lipids] (mM)	TFR (mL/min)	FRR (*v*:*v*)	Mean Size ± SD (nm)	PDI ± SD
DoE 1	3.28	4	6	877 ± 20	0.71 ± 0.01
DoE 2	3.28	4	2	3383 ± 141	0.21 ± 0.-11
DoE 3	1.10	4	6	224 ± 10	0.48 ± 0.01
DoE 4	1.10	12	6	222 ± 3	0.29 ± 0.04
DoE 5	3.28	12	2	1686 ± 55	0.93 ± 0.10
DoE 6	1.10	4	2	1157 ± 117	0.69 ± 0.01
DoE 7	2.19	8	4	308 ± 58	0.45 ± 0.11
DoE 8	3.28	12	6	266 ± 2	0.35 ± 0.01
DoE 9	1.10	12	2	1688 ± 102	0.76 ± 0.05
	*p =* 0.19	*p =* 0.40	***p =* 0.02**		

**Table 6:** Box–Behnken DoE results: neutral liposomes mean sizes and PDI (n = 3 independent experiments).

Batch Code	[Lipids] (mM)	TFR (mL/min)	FRR (*v*:*v*)	Mean Size ± SD (nm)	PDI ± SD
DoE 10	3.28	8	2	814 ± 50	0.29 ± 0.01
DoE 11	2.19	12	6	216 ± 10	0.30 ± 0.06
DoE 12	3.28	4	4	1230 ± 30	0.45 ± 0.02
DoE 13	3.28	12	4	306 ± 8	0.43 ± 0.02
DoE 14	2.19	8	4	308 ± 58	0.45 ± 0.11
DoE 15	2.19	4	6	367 ± 11	0.71 ± 0.04
DoE 16	1.10	8	2	964 ± 169	0.38 ± 0.01
DoE 17	1.10	8	6	175 ± 8	0.29 ± 0.05
DoE 18	2.19	4	2	1305 ± 152	0.49 ± 0.07
DoE 19	3.28	8	6	247 ± 58	0.60 ± 0.22
DoE 20	1.10	4	4	383 ± 1	0.48 ± 0.01
DoE 21	2.19	8	4	258 ± 6	0.45 ± 0.11
DoE 22	2.19	12	2	1484 ± 359	0.34 ± 0.07
DoE 23	1.10	12	4	289 ± 3	0.43 ± 0.02
DoE 24	2.19	8	4	358 ± 9	0.45 ± 0.11

## Data Availability

Data are contained within the article.

## Supplementary Materials

The following supporting information can be downloaded at: https://www.mdpi.com/article/10.3390/pharmaceutics16040507/s1, Table S1: Descriptive statistics of the Box-Behnken test design (batch code DOE10-DOE24).
