# Supplementary material for: Effect of Micromixer Design on Lipid Nanocarriers Manufacturing for the Delivery of Proteins and Nucleic Acids
Source: Pharmaceutics. 2024 Apr 7;16(4):507. doi: 10.3390/pharmaceutics16040507 (PMC11054535; doi:10.3390/pharmaceutics16040507)
Supplement: Supplementary file 1 [file pharmaceutics-16-00507-s001.zip › pharmaceutics-2934778-supplementary.pdf]

Table S1: Descriptive statistics of the Box-Behnken test design (batch code DOE10-DOE24)

**Source**	***p-value***
[Lipids]	0.25
TFR	0.15
FFR	**< 0.01**
[Lipids]xTFR	0.09
TFRxTFR	**< 0.05**
FFRxFFR	**< 0.05**
